# Artificial Intelligence in Anesthesia: Enhancing Precision, Safety, and Global Access Through Data-Driven Systems

**DOI:** 10.3390/jcm14196900

**Published:** 2025-09-29

**Authors:** Rakshita Giri, Shaik Huma Firdhos, Thomas A. Vida

**Affiliations:** Kirk Kerkorian School of Medicine, University of Nevada Las Vegas, 25 Shadow Lane, Las Vegas, NV 89106, USA; girir1@unlv.nevada.edu (R.G.); firdhos@unlv.nevada.edu (S.H.F.)

**Keywords:** artificial intelligence, anesthesia, closed-loop systems, robotic anesthesia, critical care, physiologic monitoring, anesthetic titration, AI-guided sedation, McSleepy, tele-anesthesia

## Abstract

Artificial intelligence (AI) enhances anesthesiology by introducing adaptive systems that improve clinical precision, safety, and responsiveness. This review examines the integration of AI in anesthetic practice, with a focus on closed-loop systems that exemplify autonomous control. These platforms integrate continuous physiologic inputs, such as BIS, EEG, heart rate, and blood pressure, to titrate anesthetic agents in real time, providing more consistent and responsive management than manual methods. Predictive algorithms reduce intraoperative hypotension by up to 40%, and systems such as McSleepy demonstrate greater accuracy in maintaining anesthetic depth and shortening recovery times. In critical care, AI supports sedation management, reduces clinician cognitive load, and standardizes care delivery during high-acuity procedures. The review also addresses the ethical, legal, and logistical challenges to widespread adoption of AI. Key concerns include algorithmic bias, explainability, and accountability for machine-generated decisions and disparities in access due to infrastructure demands. Regulatory frameworks, such as HIPAA and GDPR, are discussed in the context of securing patient data and ensuring its ethical deployment. Additionally, AI may play a transformative role in global health through remote anesthesia delivery and telemonitoring, helping address anesthesiologist shortages in resource-limited settings. Ultimately, AI-guided closed-loop systems do not replace clinicians; instead, they extend their capacity to deliver safe, responsive, and personalized anesthesia. These technologies signal a shift toward robotic anesthesia, where machine autonomy complements human oversight. Continued interdisciplinary development and rigorous clinical validation will determine how AI integrates into both operating rooms and intensive care units.

## 1. Background: Clinical Rationale and Systems-Level Potential of AI in Anesthesia

Artificial intelligence (AI) in anesthesia leverages computational power to improve patient outcomes and workflow efficiency [1,2,3]. Unlike traditional methods that depend on manual data interpretation and clinician intuition, AI provides data-driven precision through real-time analysis of large patient datasets [2,3,4]. This approach supports more accurate risk stratification, personalized anesthetic planning, and continuous intraoperative monitoring [5,6,7,8]. Machine learning algorithms reduce anesthesia-related errors compared with standard risk calculators [9]. In the postoperative setting, predictive models identify patients at risk for unplanned care escalations (UCEs), enabling earlier intervention and reducing adverse events through improved clinical decision-making [10].

**Figure 1:**
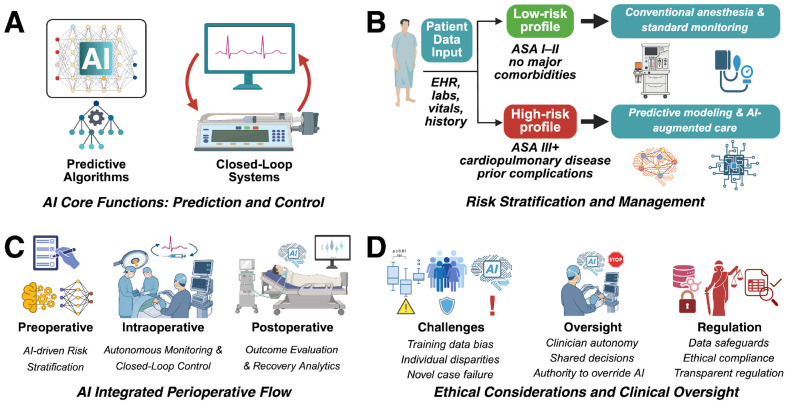
Comprehensive Integration of Artificial Intelligence in Anesthetic Care. This multi-panel schematic depicts the functional roles, clinical integration, and ethical considerations of artificial intelligence (AI) in anesthesiology. (**A**) **AI Core Functions: Prediction and Control.** Predictive algorithms analyze multidimensional patient data to generate risk profiles and guide preoperative planning. Closed-loop systems use real-time physiological inputs, such as electroencephalography (EEG)-driven indices, to adjust anesthetic drug delivery and maintain hemodynamic stability. (**B**) **Risk Stratification and Management:** AI-enhanced triage pathways incorporate data from electronic health records, laboratory results, and vital signs to classify patients as low or high risk. Low-risk patients proceed with conventional anesthesia and standard monitoring, whereas high-risk patients receive predictive modeling, preoperative optimization, and AI-augmented care strategies. (**C**) **AI Integrated Perioperative Flow:** AI is applied throughout the perioperative timeline. In the preoperative phase, machine-learning tools support individualized risk stratification. Intraoperatively, AI enables autonomous monitoring and closed-loop control. In the postoperative phase, outcome evaluation and recovery analytics inform model refinement and quality improvement. (**D**) **Ethical Considerations and Clinical Oversight:** AI introduces challenges related to data ethics and clinical governance, including bias from non-representative training data, performance disparities across populations, and reduced reliability in novel clinical scenarios. Anesthesiologists retain oversight through shared decision-making and authority to override algorithmic outputs. Regulatory frameworks ensure data protection, transparency, and accountability. This schematic serves as a conceptual synthesis of the review, functioning as a visual abstract to allow rapid comprehension of the paper’s scope and structure. ASA = American Society of Anesthesiologists physical status classification. Created using BioRender.com.

AI-driven systems improve workflow efficiency in addition to supporting direct patient care. Intelligent drug management platforms, such as automated cabinets, streamline anesthesiology department operations [11]. Logistics robots reduced the total error rate in drug and consumable distribution from 4% to 1% and saved anesthesiologists an average of 24 min per day in drug acquisition [12]. Widespread implementation of these systems requires regulatory approval, including FDA oversight and institutional policy alignment, to ensure patient safety. Figure 1 provides a conceptual overview of how AI integrates across anesthetic care, spanning prediction, control, and ethical oversight.

## 2. Objective

This review examines the integration of artificial intelligence (AI) into anesthesia, focusing on its ability to improve precision, safety, and efficiency in anesthetic care. It explores AI applications across the perioperative continuum, including preoperative planning, intraoperative monitoring, and postoperative management, with emphasis on individualized, data-driven patient care. Current implementations and emerging technologies are reviewed alongside the evolving role of anesthesiologists, highlighting how AI augments decision-making, streamlines workflows, and strengthens interdisciplinary communication. Broader implications for patient outcomes include fewer complications, faster recovery, and improved access in resource-limited health systems. The review also addresses challenges such as algorithmic bias, regulatory oversight, and the need for clinician education to ensure safe and effective use. Overall, AI functions as an adjunct that supports, rather than replaces, anesthesiologists, with implications extending beyond anesthesia to modern medicine.

## 3. Evolution of AI in Anesthesia

The development of artificial intelligence (AI) in anesthesia has evolved over several decades. In the 1990s, decision-support systems were integrated into anesthesia monitoring through patient data management systems (PDMS), which captured physiologic data from operating room devices. These systems provided alerts for deviations from preselected physiologic or technical values and later incorporated algorithm-based decision support [13]. While useful, early systems offered only basic alerts without predictive capabilities.

By the 2010s, machine learning and deep learning enabled more advanced applications, including predictive modeling and automated anesthesia delivery. Closed-loop platforms use parameters such as the bispectral index (BIS) or wavelet-based anesthetic value for central nervous system monitoring (WAVCNS) to adjust infusion rates in real time. One WAVCNS-based system successfully controlled propofol and remifentanil administration with improved performance and safety [14]. In cardiac surgery, another closed-loop platform maintained total intravenous anesthesia without manual intervention in 80% of cases [15]. These systems stabilize anesthetic depth, reduce manual adjustments, and decrease intraoperative hypotension events by up to 40% [16].

Despite these advances, AI remains limited in complex surgeries where unpredictable changes in patient condition or drug response occur. Traditional approaches, though less precise, benefit from the anesthesiologist’s ability to rapidly adapt to unforeseen events. As a result, the anesthesiologist’s role is shifting from manual drug administration to system oversight and intervention. In this collaborative model, AI augments rather than replaces human expertise [17].

Safe adoption requires continued regulatory evaluation. Agencies such as the FDA assess these technologies for safety and efficacy before clinical integration. Ethical, legal, and practical issues—including algorithmic bias, accountability for adverse outcomes, and data privacy—remain critical challenges. Addressing these concerns will be essential to ensure equitable and responsible use of AI in anesthesia. The Sedasys system, while demonstrating safety and efficacy in controlled settings, faced challenges beyond cost and market uptake. Its use was restricted to low-risk patients (ASA I-II), with airway emergencies and complex cases excluded. Concerns about patient safety, anesthesiologist resistance, and medico-legal liability contributed to its poor adoption. Regulatory hesitations and the absence of reimbursement pathways further limited uptake, ultimately leading to its withdrawal from the market despite FDA approval.

### 3.1. AI Enables Scalable Anesthesia Support in Low-Resource Health Systems

AI can help address anesthesiologist shortages in low-resource settings, where limited access to trained providers contributes to elevated perioperative mortality rates [18]. AI-driven tele-anesthesia systems allow experienced clinicians to remotely supervise procedures in rural or underserved hospitals [19]. These platforms integrate real-time monitoring, predictive analytics, and automated drug delivery to support local staff and reduce the strain on limited personnel [20].

In sub-Saharan Africa, AI-supported remote monitoring has guided non-specialist providers through surgical care [19]. Cloud-based algorithms interpret vital signs and recommend anesthetic adjustments, improving perioperative safety without requiring full-time anesthesiologists on-site.

Despite this potential, implementation challenges persist. Many AI platforms require high-speed connectivity, robust infrastructure, and significant capital investment, resources often unavailable for developing health systems [21]. Regulatory approval processes, typically designed for high-income countries, may not align with local governance structures, further complicating implementation [22].

### 3.2. Equitable AI Integration Requires Open Access and Global Coordination

Equitable adoption of AI in anesthesia depends on investment, accessibility, and global collaboration. International organizations and policymakers can reduce the technological divide between high- and low-income regions by supporting affordable and adaptable infrastructure [23]. Open-source platforms provide cost-effective systems that allow local customization without reliance on proprietary tools [24]. Partnerships among governments, NGOs, and technology firms can subsidize deployment in underserved areas and expand access beyond well-resourced health systems [25]. The core applications of AI in anesthesia and their benefits are summarized in Table 1.

## 4. Clinical Applications and Evidence

### 4.1. Preoperative Applications

AI plays an increasing role in preoperative assessments, using advanced machine-learning algorithms to analyze large patient datasets and predict surgical risks [27]. Traditional evaluations rely on clinician judgment, which can vary between providers. In contrast, AI models integrate medical history, genetic data, vital signs, and social factors to generate individualized anesthesia plans [28]. Predictive models such as MySurgeryRisk, which use electronic health records (EHRs) to identify postoperative complications, outperform human assessments in predicting outcomes including cardiovascular complications and respiratory distress [29]. These models detect subtle patterns that may be overlooked in routine evaluations, such as minor variations in baseline vital signs or genetic predispositions. By forecasting anesthesia-related risks, including allergic reactions, airway difficulties, and adverse drug interactions, AI allows anesthesiologists to prepare more effectively for complications. This data-driven approach supports precision medicine in anesthesiology, where patient care is tailored in real time using comprehensive and continuously updated datasets. While multiple artificial intelligence (AI) applications demonstrate promising roles across the perioperative pathway, the strength and quality of supporting evidence vary considerably. Some systems, such as closed-loop anesthetic controllers, have been tested in randomized clinical trials, whereas others, including predictive models for complications and tele-anesthesia platforms, remain at pilot or proof-of-concept stages. To provide a clear appraisal, Table 2 summarizes the current evidence base, distinguishing study types, principal findings, and key limitations for each major AI application in anesthesiology. While closed-loop systems such as McSleepy have demonstrated promising performance in controlled environments, evidence from real-world clinical practice remains limited. Studies suggest variability in system performance across different patient populations, with greater reliability in low-risk cohorts than in patients with significant comorbidities or complex surgical profiles. These findings highlight the importance of broader validation before widespread adoption can be considered.

This table summarizes the current evidence base for major AI applications in anesthesiology and highlights the type of study design, principal findings, and key limitations. The appraisal distinguishes between randomized trials, observational studies, and proof-of-concept pilots, underscoring both the promise and the constraints of these technologies.

### 4.2. Intraoperative Applications

AI-driven systems such as McSleepy and Sedasys automate anesthetic delivery through closed-loop control. These platforms continuously monitor vital signs, heart rate, blood pressure, and oxygen saturation, and adjust anesthetic dosing to maintain target depth of anesthesia. Machine-learning algorithms trained on patient response data enable real-time titration during surgery. The Sedasys system received FDA approval to autonomously administer propofol for routine endoscopic procedures, reducing the need for continuous anesthesiologist supervision [30]. In a multicenter randomized trial, Sedasys lowered the area under the oxygen desaturation curve compared with benzodiazepine/opioid sedation (23.6 s·% vs. 88.0 s·%; *p* = 0.028), and patients reported higher satisfaction, faster recovery, and fewer adverse events (5.8% vs. 8.7%) [30].

Despite these benefits, Sedasys illustrates the limitations of AI in anesthesia. The system was designed for mild-to-moderate sedation and cannot manage deep sedation required for complex cases [32]. It also lacks the capacity to address airway and ventilation complications, which demand immediate clinical intervention [32].

Additional challenges include reliance on non-anesthesiologist personnel, who may not have the expertise to manage complications [30], and limited adoption due to high costs and poor market acceptance, which led to its withdrawal [33]. Patients with comorbidities or undergoing high-risk procedures often require nuanced adjustments and responses to unanticipated drug interactions or physiological changes that current AI systems cannot provide [34].

These limitations emphasize that AI enhances safety and efficiency in routine procedures but cannot replace anesthesiologist oversight in high-stakes situations. AI should be regarded as a supportive tool that augments, rather than substitutes, clinician expertise (Table 3).

AI-based real-time patient monitoring supports anesthesiology by integrating applications such as depth-of-anesthesia tracking, anesthetic control, event prediction, and operating room logistics [2]. Predictive algorithms analyze large datasets—including heart rate variability, respiratory patterns, and other physiologic markers—to provide anesthesiologists with decision support [36]. For example, models can forecast intraoperative events such as hypotension or hypoxemia, enabling earlier intervention [37].

Electroencephalogram (EEG)-based systems continuously assess anesthetic depth by generating indices that guide dosing adjustments and improve outcomes [38]. These tools are especially valuable in high-risk procedures, where small physiologic changes may lead to significant complications. In cardiac surgery, AI algorithms monitor blood pressure, heart rate, and oxygen saturation in real time to identify subtle anomalies before they progress [39]. In neurosurgery, AI assists in maintaining optimal anesthesia levels, reducing the risk of both over-sedation and under-sedation [40]. Machine-learning models trained on extensive perioperative datasets predict complications such as hypotension, hypoxia, and arrhythmias earlier than clinical recognition. These predictions support targeted interventions, including drug titration or fluid resuscitation, and reduce the likelihood of adverse outcomes [41]. By synthesizing data from multiple monitoring devices, AI provides anesthesiologists with a comprehensive view of the patient’s status, reducing cognitive load and allowing greater focus on complex decision-making [42]. While AI enhances monitoring and safety, anesthesiologists remain essential. Clinical judgment is required to interpret context, select interventions, and coordinate with the surgical team. AI functions as a decision-support tool that augments, but does not replace, the expertise of the anesthesiologist.

These enhancements in monitoring and prediction have translated into measurable clinical benefits. Comparative studies show that AI-driven anesthesia platforms match or outperform manual techniques in several domains, including depth-of-anesthesia control, drug titration accuracy, and complication rates. These findings, drawn from retrospective analyses and semi-prospective trials, are summarized in Table 4.

Beyond measurable improvements in anesthetic control and complication rates, AI also strengthens perioperative teamwork by enhancing communication and supporting coordinated responses to patient risk. These systems not only improve efficiency but also create a shared, data-driven framework that links anesthesiologists, surgeons, and nursing staff throughout the surgical process.

AI enhances communication among anesthesiologists, surgeons, and other providers by centralizing patient data and presenting real-time, objective insights accessible to the entire surgical team. In complex surgeries where coordination is critical, AI platforms collect, process, and display patient vitals, anesthetic depth, and other parameters in a shared interface. This ensures all team members have access to the same up-to-date information, lowering the risk of errors from inconsistent communication.

AI further strengthens communication by supplementing or replacing traditional verbal handoffs, which are often prone to misinterpretation or omission [47]. During surgery, AI systems can automatically generate alerts when patient status changes, such as oxygen desaturation or arrhythmias. These updates, delivered via monitors or integrated platforms, provide immediate, standardized notifications to the entire surgical team.

Beyond communication, AI’s predictive analytics extend this collaborative framework by enabling proactive rather than reactive management. Machine-learning models using preoperative and intraoperative data can forecast intraoperative bradycardia associated with hypotension with an AUC of up to 0.89 [35]. Other models predict post-induction hypotension with AUC values as high as 0.76 [48]. By anticipating complications before they become clinically apparent, these tools allow the surgical team to implement preemptive measures in a coordinated manner, avoiding fragmented responses that could compromise patient safety.

### 4.3. Postoperative Applications

AI improves postoperative communication by generating detailed, standardized reports that summarize intraoperative events, anesthetic dosing, and anticipated risks. These structured outputs improve the quality of handoffs between anesthesia providers, recovery room staff, and intensive care teams, ensuring continuity of care. For example, machine-learning models trained on the APRICOT dataset classified pediatric patients as low risk for severe perioperative critical events with high accuracy and negative predictive value, highlighting their utility for postoperative triage and monitoring [49].

In addition, AI-driven predictive models can identify patients at risk for complications such as hypotension or hypoxemia during recovery, enabling early interventions in the post-anesthesia care unit (PACU). By centralizing and communicating these risk assessments, AI supports coordinated, proactive management across the postoperative team and reduces preventable adverse outcomes [37].

### 4.4. Comparative Outcomes

Comparative studies consistently demonstrate that AI-driven anesthesia can equal or surpass manual practice across several performance domains. For example, AI systems maintained target BIS values 75–89% of the time, compared with 56–60% for manual approaches [43]. Propofol titration guided by AI reduced performance errors (MDPE −1.1% vs. −10.7%; MDAPE 9.1% vs. 15.7%) and decreased the need for manual interventions (8 vs. 22 per case) [43,44] Intraoperative complication rates were also lower with AI systems (17% vs. 36%) [45].

In predictive modeling, ensemble machine-learning algorithms such as XGBoost (AUC = 0.95), Gradient Boosting (AUC = 0.912), and Random Forest (AUC = 0.842) outperformed manual clinical assessment in forecasting postoperative outcomes, including acute kidney injury, hypotension, and mortality [45]. These findings add to the evidence that AI can strengthen clinical decision-making and improve perioperative outcomes when properly validated and integrated into practice.

However, most existing studies were conducted under controlled conditions with select surgical populations. Broader validation in diverse, real-world environments is needed to confirm generalizability and determine the practical role of AI in routine perioperative care. Stronger evidence will also inform perioperative planning and resource allocation strategies.

### 4.5. Role of Anesthesiologists in AI-Supported Care

The rise of AI in anesthesia is shifting the role of anesthesiologists from direct monitoring to supervision. As AI systems take over repetitive, data-driven tasks such as tracking vital signs and adjusting anesthetic doses, anesthesiologists focus on complex aspects of care that require intuition, critical thinking, and ethical judgment [50]. They oversee AI platforms, troubleshoot when necessary, and intervene during unexpected complications.

This shift creates both opportunities and challenges. Anesthesiologists gain time to address aspects of care that require human expertise, such as interpreting non-verbal cues, recognizing emotional states, and making ethical decisions. At the same time, the evolving role demands new skills, including proficiency with AI technologies and the ability to integrate machine outputs with clinical judgment [51]. Anesthesiologists must interpret AI findings, identify system errors, and intervene when limitations are reached. The future of anesthesia care will depend on a collaborative relationship in which clinicians and AI systems contribute complementary capabilities.

## 5. Limitations and Risks

### 5.1. Technical and Clinical Limitations

AI manages routine anesthetic tasks effectively but continues to face limits in complex or emergency settings. Systems such as Sedasys performed well in low-risk procedures like routine endoscopies but were less effective in patients with comorbidities or unpredictable reactions [32,34]. Advances may eventually allow adaptive, real-time dosing based on individual responses [52], but current systems lack the flexibility and contextual awareness of human clinicians. Anesthesiologists remain essential for addressing complications, managing rapid physiologic changes, and exercising judgment in situations beyond algorithmic capability.

### 5.2. Limitations of Model Transferability

Beyond clinical validation, algorithm development itself faces substantial challenges. Many systems demonstrate limited generalizability when applied to patient populations or surgical contexts that differ from their training data. Robustness in rapidly changing or high-complexity clinical environments remains a critical concern, as algorithms may fail under edge-case conditions or when confronted with unanticipated physiologic perturbations. These limitations underscore the need for cautious implementation and ongoing refinement of AI systems.

### 5.3. Ethical and Legal Risks

Accountability in AI-driven anesthesia remains a key ethical concern. Anesthesiologists have traditionally held full responsibility for patient safety, but liability becomes more complex when AI contributes through predictive analytics, automated drug delivery, or intraoperative monitoring [53]. If an AI system provides a recommendation that a physician follows and an adverse outcome occurs, the physician may still be held liable if they failed to apply appropriate clinical judgment or if the recommendation deviated from accepted standards of care [54].

Discussions in medical ethics and law suggest that responsibility in AI-administered anesthesia may extend to multiple stakeholders, including software developers, hospital systems, and the supervising anesthesiologist [55]. Legal frameworks are beginning to address AI accountability, but definitive precedents remain limited. Traditional liability models, such as fault-based and strict liability, may not fully apply to dynamic AI systems. In conventional anesthetic practice, liability is typically fault-based: a medical professional may be held accountable for negligence or deviation from the standard of care if an error, such as administering an incorrect dosage or failing to monitor vital signs, directly causes harm. Establishing liability requires demonstrating duty, breach, causation, and damages, making each case context-specific.

Strict liability holds a party responsible for harm regardless of fault or intent. Applied to anesthesia, this would mean that if an AI-driven system caused harm, such as delivering an incorrect anesthetic dose, the hospital, software developer, or manufacturer could be liable even without negligence [56]. In contrast, fault-based liability requires proving that the AI directly caused the harm. AI-assisted anesthesia complicates this distinction because anesthesiologists continue to make clinical decisions. If an AI issues an incorrect recommendation and the anesthesiologist follows it without independent verification, responsibility may be attributed to either the clinician or the AI developer. Under strict liability, manufacturers and developers could face lawsuits simply because their technology was involved in an adverse event [56]. This uncertainty, along with the principal–agent model that places responsibility on physicians for AI decisions, may discourage adoption of these tools [54].

Ethical and legal challenges may significantly influence the adoption of AI in routine anesthetic care. Algorithmic bias and lack of explainability raise concerns about fairness and transparency, particularly in high-stakes settings where clinicians must justify decision-making. Medico-legal accountability represents another unresolved issue: it is unclear whether responsibility for adverse outcomes rests with clinicians, institutions, or developers when AI-generated recommendations are followed. These concerns highlight that technical performance alone will not determine adoption; trust, governance, and clear regulatory frameworks will be equally decisive.

Beyond legal ambiguity, the evidence base for many AI applications in anesthesiology remains limited. As outlined in Table 2, many AI applications in anesthesiology remain constrained by small sample sizes, limited real-world validation, and validation and variable generalizability, underscoring the need for cautious interpretation and further large-scale studies.

These evidence gaps are especially apparent in complex or emergent cases. Closed-loop systems such as Sedasys automate sedation effectively in healthy patients undergoing routine procedures but have difficulty managing patients with comorbidities, deep sedation requirements, or sudden physiologic instability [32]. In such situations, nuanced clinical judgment remains essential. Experienced anesthesiologists recognize subtle patterns, reassess patient status, and adjust management in ways AI does not yet match. AI should therefore be regarded as a supportive tool rather than a replacement. Safe implementation requires systems with fail-safes and clear triggers for clinician override in unpredictable clinical environments. Adoption of AI in anesthesiology also faces resistance from within the profession. Concerns include potential job displacement, medico-legal liability if AI-driven care leads to adverse outcomes, and skepticism about the reliability of algorithmic recommendations in unpredictable clinical contexts. Training gaps further compound this resistance, as many anesthesiologists have limited exposure to AI systems in their formal education.

### 5.4. Bias and Equity Concerns

AI’s effectiveness in anesthesia depends on the quality and diversity of the datasets used to train its algorithms. Models built on electronic health records (EHRs) may reflect algorithmic, implicit, or selection biases that risk worsening healthcare disparities [57]. For example, anesthesia monitoring tools calibrated mainly on Western patient populations may perform less accurately in underrepresented groups, potentially affecting dosing precision and complication rates [57]. Strategies for mitigating these biases include data preprocessing techniques like resampling and reweighting, which aim to create more balanced datasets.

Mitigating these risks requires the use of diverse, representative datasets in AI model development and greater transparency in algorithmic decision-making [58]. Regular auditing and bias detection frameworks should be applied to ensure equitable performance across patient populations. Without these safeguards, AI-driven anesthesia could perpetuate rather than reduce existing healthcare disparities.

### 5.5. Data Privacy and Cybersecurity

Integrating AI into anesthesiology relies on large volumes of patient data for decision-making and predictive analytics. This dependence raises concerns about patient consent, data ownership, and security risks [59]. Traditional informed consent models may be inadequate, as patients are often unaware of how their data contribute to AI development. In addition, the legal ownership of AI-generated insights remains unclear, underscoring the need for updated regulatory frameworks.

AI systems in anesthesiology also pose significant cybersecurity vulnerabilities, with cloud-based storage and real-time monitoring increasing the risk of data breaches. Healthcare institutions remain prime targets for cyberattacks, and breaches of AI-driven anesthesia platforms could compromise patient safety [60]. Robust encryption, federated learning, and blockchain-based audit trails have been proposed to mitigate these risks.

For AI-driven anesthesia systems, compliance with regulations such as HIPAA and GDPR helps ensure secure data handling, ethically sourced training datasets, and institutional accountability. These safeguards reduce the risk of data breaches, biased algorithms, and ethical violations. Unlike traditional healthcare data management, however, AI platforms continuously collect and analyze large volumes of patient information, creating challenges for maintaining data security, de-identification, and access control.

### 5.6. Anesthesiologist Resistance

Despite the potential benefits of AI in anesthesia, many anesthesiologists express resistance to its widespread adoption. Job security is a central concern, with some clinicians perceiving AI as a threat that may reduce the need for anesthesiologists in routine, low-risk procedures. Medico-legal liability adds further hesitation, as accountability remains unclear when errors occur in AI-assisted care. Clinicians may worry about being held responsible for adverse outcomes even when decisions are influenced by algorithmic recommendations.

Training gaps also contribute to resistance. Many anesthesiologists lack formal education in AI principles, data interpretation, or system troubleshooting, leaving them uncertain about how to oversee these technologies effectively. Without structured AI training in residency programs and continuing education, adoption may remain slow. Addressing these concerns through transparent role definitions, clear liability frameworks, and targeted education will be critical for building clinician trust and supporting responsible AI integration.

### 5.7. Autonomy and Informed Consent

AI integration into anesthesia also complicates the traditional consent process. Patients must now be informed about AI’s role in their care, autonomy level, and its limitations [61]. An ethically sound consent process should account for AI’s level of autonomy, its potential deviation from clinical norms, and associated risks [62]. Clinicians should also be able to explain how the system functions, its validation status, and known biases to ensure patient understanding and maintain trust [63].

Many patients are unfamiliar with AI in healthcare and may express concern about its role in critical decision-making. Some may prefer to decline AI-assisted anesthesia, but current systems do not always allow this, particularly in urgent settings.

Studies indicate that education materials and structured consent forms describing AI involvement help maintain patient trust [62,64]. As AI becomes more common in anesthetic practice, institutions should emphasize transparent communication to support patient autonomy and ethical standards. Table 5 summarizes key challenges associated with AI-driven anesthesia.

Beyond informed consent, patient perspectives are central to the ethical integration of AI in anesthesiology. Surveys suggest that many patients express greater comfort when clinicians remain in the loop, reflecting concerns about depersonalized care and algorithmic autonomy. Trust, transparency, and clear communication about AI’s role will be essential for patient acceptance.

## 6. Future Directions

### 6.1. Education and Training

The traditional anesthesiology curriculum, which emphasizes pharmacology, physiology, and hands-on procedural training, should also incorporate AI literacy. Future anesthesiologists need familiarity with machine learning principles, data analytics, and algorithmic bias [65]. Fundamental AI education should include coursework on data interpretation, troubleshooting system errors, and assessing algorithmic recommendations within a clinical context.

In addition to theoretical knowledge, hands-on AI experience is important for developing proficiency. Simulation-based training, where trainees interact with AI-driven anesthesia monitoring systems in controlled environments, increases familiarity with AI-assisted decision-making [66]. These simulations allow residents to evaluate AI predictions, identify errors, and practice appropriate interventions. Incorporating AI training into residency programs supports preparedness for technological integration while reinforcing patient safety and clinical judgment.

AI can improve anesthesia management, but its limitations require continuous human oversight. Anesthesiologists should evaluate AI outputs critically rather than accept them unconditionally. Studies show that overreliance on AI may lead to automation bias, where clinicians follow recommendations without verification and increase the risk of errors [67].

Training programs should emphasize cognitive resilience and decision-making frameworks that account for AI fallibility. Case-based learning, where residents review real-world AI failures in anesthesia, can strengthen their ability to identify algorithmic shortcomings. Interdisciplinary collaboration among anesthesiologists, data scientists, and engineers can also improve understanding of AI functionality and limitations [68]. A critical approach to AI use positions these systems as decision-support tools rather than replacements for clinical expertise.

Educational initiatives integrating AI into anesthesiology training are emerging. Pilot programs using simulation-based platforms and dedicated AI modules in residency curricula illustrate potential pathways for adoption. Addressing these training gaps may not only enhance clinician preparedness but also mitigate resistance to AI integration.

Although AI use in anesthesia is expanding, maintaining fundamental anesthetic skills remains essential. Closed-loop drug delivery and predictive monitoring may reduce the frequency of manual interventions, but reduced practice risks skill atrophy, especially when AI systems fail or are unavailable [69].

To counteract this, anesthesia training must incorporate structured skill-retention strategies. Regular manual anesthetic administration rotations, where residents practice drug titration and airway management without AI assistance, help preserve technical proficiency. Periodic assessments should evaluate clinicians’ ability to shift between AI-assisted and conventional anesthesia. Competency without AI support is essential for managing emergencies that demand rapid, intuitive decision-making [17].

A culture of continuous learning supports safe AI integration as technologies evolve. Residency programs should include professional development opportunities such as AI-focused workshops and retraining to keep anesthesiologists proficient in both AI collaboration and independent clinical practice [66]. Balancing AI adoption with skill preservation ensures preparedness while maintaining high standards of patient care.

### 6.2. Global Health and Tele-Anesthesia

Applications of AI in global health, including tele-anesthesia and remote monitoring, remain largely at the proof-of-concept stage. Significant infrastructural barriers—such as unreliable internet connectivity, inconsistent power supply, and limited access to physiologic monitoring equipment—present substantial obstacles. Workforce training requirements and disparities in access further complicate implementation in low- and middle-income countries. Without deliberate strategies to address these inequities, AI may risk widening rather than narrowing global gaps in anesthetic care.

Tele-anesthesia and remote AI-assisted monitoring represent an emerging area of application. In underserved regions with limited access to anesthesiologists, these systems allow off-site specialists to support local providers. AI-enabled platforms can monitor vitals, adjust drug levels, and flag anomalies in real time to improve care in low-resource settings. Pilot programs in Kenya and India demonstrated reductions in perioperative complications and improvements in surgical safety [70]. Barriers to broader implementation include limited internet connectivity [71], a shortage of AI-trained clinicians [51], high hardware costs, and inadequate policy infrastructure [41,72].

Partnerships between governments, non-government organizations (NGOs), and private technology firms can support infrastructure development, subsidize implementation, and expand training pipelines [73]. AI education should be integrated into undergraduate medical curricula and anesthesia residency programs, including training in algorithm interpretation, ethical use, and patient communication. For practicing clinicians, continuing education modules and international knowledge-sharing networks can help reduce the skills gap [2].

### 6.3. Regulatory and Policy Evolution

AI’s role in anesthesiology will continue to expand, integrating more deeply into preoperative assessments, intraoperative monitoring, and postoperative care. This expansion raises essential questions about the training of future anesthesiologists, the regulation of AI-driven decision-making and the impact on patient outcomes. By embracing AI while maintaining human oversight, the field can harness the full potential of this technology while ensuring ethical and patient-centered care. Additionally, policy frameworks must evolve alongside AI advancements, incorporating updated approval processes and hospital guidelines to ensure responsible AI integration. Furthermore, expanding tele-anesthesia initiatives and reducing disparities in AI accessibility will be critical in ensuring that all healthcare systems, regardless of economic status, can benefit from these advancements.

The application of AI in global health and tele-anesthesia remains at an early, proof-of-concept stage. While pilot programs highlight feasibility, significant barriers exist, including limited digital infrastructure, workforce training deficits, and ethical challenges in low-resource contexts. These factors underscore the need for cautious framing rather than assuming widespread scalability in the near term.

As the field evolves, regulatory frameworks must evolve alongside AI adoption. National and international bodies should set AI validation, integration, and oversight standards specific to perioperative care. Priorities include transparency in algorithm design, clear performance benchmarks, and accountability for errors [71]. Institutions should also establish pathways for safe implementation and for responding to unexpected failures.

Partnerships between governments, non-government organizations (NGOs), and private technology firms can support infrastructure development, subsidize implementation, and expand training pipelines [2]. AI education should be integrated into undergraduate medical curricula and anesthesia residency programs, including training in algorithm interpretation, ethical use, and patient communication. For practicing clinicians, continuing education modules and international knowledge-sharing networks can help reduce the skills gap [72].

### 6.4. Toward an Equitable and Collaborative Future

AI will not replace anesthesiologists but will redefine their practice. The specialty’s future lies in collaborative integration, where clinicians and AI contribute complementary strengths. In high-stakes settings—such as surgeries involving major blood loss or hemodynamic instability—AI provides real-time data analysis and alerts, while anesthesiologists apply clinical judgment to interpret complex, non-quantifiable factors like emotional state or unexpected drug responses [46]. Combining computational capacity with human expertise enhances patient safety, improves care quality, and reduces routine workload.

Responsible integration will depend on transparency, equity, and shared oversight. Expanding access to AI-supported anesthesia in under-resourced health systems requires inclusive design, context-sensitive implementation, and policies that prioritize patient safety across diverse populations. These principles should guide the specialty as it adapts to an increasingly data-driven era.

## 7. Conclusions

Artificial intelligence is reshaping anesthesiology by improving precision and efficiency, while expanding rather than replacing the role of anesthesiologists. As these technologies embed more deeply into perioperative workflows, the field must prioritize ethical and practical challenges, including accountability, algorithmic bias, data privacy, and equitable access.

Preparing clinicians for this transition requires balancing traditional anesthetic skills with AI literacy, simulation-based training, and safeguards against automation bias and skill atrophy. Ongoing professional development will ensure that practicing anesthesiologists remain proficient in both AI-enabled and conventional practice.

Equally important, policy frameworks and institutional guidelines must evolve to ensure transparent validation, reliable oversight, and protection of patient data. Future research should focus on building trust through human-in-the-loop models that preserve physician oversight while enhancing decision support.

Finally, expanding global initiatives such as tele-anesthesia will demand investment in infrastructure and partnerships among governments, non-governmental organizations, and private technology firms. While AI holds considerable promise as an augmentative force in anesthesiology, its safe and equitable integration into practice will require rigorous validation in diverse, real-world clinical environments. Success will depend on addressing professional and patient concerns, building robust governance frameworks, and ensuring that ethical, legal, and infrastructural challenges are not overlooked. Only with this balanced approach can AI truly advance precision, safety, and equity in anesthetic care.

## Figures and Tables

**Table 1:** Core AI Applications in Anesthesia and Associated Benefits.

AI Application	Function	Benefit
Predictive Modeling [26]	Identifies surgical risks using patient data	Reduces anesthesia-related errors
Closed-Loop Anesthesia Systems [14]	Automates anesthetic drug administration	Improves stability, reduces hypotension
Machine Learning-Based Risk Assessment [25]	Predicts complications (hypotension, hypoxia)	Allows proactive interventions
AI-Driven Inventory Management [26]	Automates drug and supply tracking)	Reduces distribution errors from 4% to 1%

**Table 2:** Evidence Strength of AI applications in Anesthesiology.

AI Application	Study Type	Function	Benefit
Sedasys System (computer-assisted propofol sedation) [30]	Multicenter randomized control trial (≈1000 patients, FDA-approved trial, U.S.)	Demonstrated safe delivery of moderate propofol sedation in low-risk patients; reduced anesthesiologist presence at bedside; comparable safety outcomes to conventional care.	Restricted to ASA I-II patients; excluded airway emergencies; not adaptable in complex cases; professional resistance; lack of reimbursement pathways; withdrawn from market despite FDA approval.
McSleepy (closed-loop total intravenous anesthesia system) [30]	Pilot studies (single-center, <100 patients)	Automated propofol-remifentanil delivery achieved stable hemodynamics and adequate anesthesia; feasibility demonstrated.	Very small studies; limited generalizability; not validated in diverse or high-risk populations; requires specialized hardware.
Closed loop anesthetic depth control (EEG-based BIS monitoring) [14]	Single-center RCTs (50–150 patients each)	Improved anesthetic depth stability; reduced anesthetic consumption; shortened emergence vs. manual titration.	Mostly controlled trial settings; small sample sizes; external validation lacking; not widely adopted in real-world OR environments.
Predictive analytics for intraoperative hypotension (e.g., Hypotension Prediction Index) [31]	Observational studies + pilot trials	Predicts intraoperative hypotension minutes in advance with high AUC (>0.85); potential to allow earlier intervention.	Requires invasive arterial monitoring; prone to false positives; performance may degrade in novel populations; not yet integrated into standard practice.
Machine learning for postoperative complications (e.g., AKI, delirium, ICU transfer) [5]	Retrospective observational studies (electronic health record datasets)	Achieved high predictive accuracy (AUC > 0.80) for risk stratification.	Trained on retrospective data; not prospectively validated; limited external generalizability; risk of overfitting.
Tele-anesthesia and AI-guided anesthesia in low-resource settings [18]	Proof-of-concept reports; pilot implementations	Demonstrated feasibility of remote monitoring and AI-assisted monitoring and AI-assisted sedation in select LMIC contexts; potential to extend anesthesia services.	Very limited evidence; infrastructure gaps; clinician training barriers; high variability in implementation feasibility; ethical/legal frameworks underdeveloped.

**Table 3:** a. Comparison of AI Anesthesia Systems. b. Performance Data for AI vs. Traditional Anesthesia Administration.

a			
Feature	Sedasys	McSleepy	Manual Administration
**AI Involvement**	Closed-loop, ML-based drug delivery	Automated propofol delivery via feedback loop	Human-guided drug titration
**Primary Function**	Maintain anesthesia depth during surgery	Mild-to-moderate sedation for endoscopy	Flexible anesthetic management across case types
**Clinical Approval**	Research prototype	FDA-approved (withdrawn) [30]	Gold standard; no special approval required
**Effectiveness in Routine procedures**	High in controlled environments [15]	High—Reduced desaturation and faster recovery [30]	Effective when administered by trained anesthesiologists [17]
**Effectiveness in Complex Cases**	Limited [15]	Not suitable for deep sedation or airy issues [31]	High adaptability for complex scenarios [17]
**Anesthesiologist Supervision Required**	Yes	No—operated by non-anesthesia personnel [30]	Yes
**Risk of Complications**	Low in simple cases [15]	Higher risk if unexpected complications arise [32]	Generally low with skilled providers [17]
**Market Adoption**	Experimental use in academic settings	Poor—Withdrawn due to low market uptake [33]	Universal
**Limitations**	Limited real-world scalability; lacks validation [15]	Cannot manage deep sedation; lacks responsiveness to emergencies [32,33]	Labor-intensive; subject to inter-provider variability
**b.**			
**Methods**	**Outcome**	**AI Performance**	**Traditional Method Performance**
**Sedasys System (Propofol)**	Maintenance of oxygen saturation	Reduced desaturation events (74%) [30]	Higher rate of desaturation events
**Closed-Loop TIVA in Cardiac Surgery**	Automated anesthesia delivery	80% of cases completed without manual intervention [15]	Manual adjustments required throughout
**Hypotension Prediction Models**	Prediction of intraoperative hypotension	89% accuracy in detecting bradycardia associated hypotension [35]	Traditional risk assessment methods are less precise

**Table 4:** Comparison of AI-driven versus manual anesthesia management across key performance domains.

Category	AI-Driven Management	Manual Management	Key Findings
Depth of Anesthesia (BIS)	Maintained BIS 40–60 in 75–89% of time	Maintained BIS 40–60 in 56–60% of time	Significantly improved depth control with AI
Performance Error	MDPE—1.1%, MDAPE 9.1%	MDPE—10.7%, MDAPE 15.7%	More accurate propofol titration with AI [43]
Induction to EtAA Target	Median time to target: 75 s	Median time: 158 s	Faster anesthetic onset with AI [44]
Manual Interventions	8 adjustments per case	22 adjustments per case	Reduced intervention burden with AI [45]
Moderate/Major Complications	17% rate	36% rate	Lower complication rates with AI [45]
Predictive Accuracy	XGBoost AUC 0.95; Gradient Boosting AUC 0.912; Random Forest AUC 0.842	No formal predictive model	ML models outperform manual judgment for AKI prediction [46]

Note: “AI-driven management” refers to closed-loop anesthesia delivery systems that use real-time physiological monitoring (e.g., Bispectral Index [BIS]) and control algorithms to adjust anesthetic drug dosing automatically. These systems typically control agents such as propofol or volatile anesthetics via feedback loops to maintain the target depth of anesthesia.

**Table 5:** Ethical Challenges in AI-Driven Anesthesia.

Ethical Challenge	Description	Potential Solution
**Algorithmic Bias**	AI models trained on biased datasets may lead to disparities in anesthesia management	More diverse training datasets, bias audits
**Liability and Accountability**	Determining legal responsibilities for AI errors	Shared accountability models, regulatory frameworks
**Data Privacy Risks**	AI requires vast amounts of patient data	Strong encryption, federated learning

## Data Availability

No new data were created or analyzed in this study.

## Abbreviations

The following abbreviations are used in this manuscript:

AI	Artificial Intelligence
BIS	Bispectral Index
EEG	Electroencephalography
EHR	Electronic Health Record
FDA	U.S. Food and Drug Administration
GDPR	General Data Protection Regulation
HIPAA	Health Insurance Portability and Accountability Act
MDPE	Median Performance Error
ML	Machine Learning
PDMS	Patient Data Management System
TIVA	Total Intravenous Anesthesia
UCE	Unplanned Care Escalation
WAVCNS	Wavelet-based Anesthetic Value for Central Nervous System
